# Evaluation of Patient and Medical Staff Satisfaction regarding Healthcare Services in Wuhan Public Hospitals

**DOI:** 10.3390/ijerph15040769

**Published:** 2018-04-17

**Authors:** Runtang Meng, Jingjing Li, Yunquan Zhang, Yong Yu, Yi Luo, Xiaohan Liu, Yanxia Zhao, Yuantao Hao, Ying Hu, Chuanhua Yu

**Affiliations:** 1 Department of Preventive Medicine, School of Health Sciences, Wuhan University, 185 Donghu Road, Wuhan 430071, China; mengruntang@whu.edu.cn (R.M.); Yun-quanZhang@whu.edu.cn (Y.Z.); huy@whu.edu.cn (Y.H.); 2 Department of Behavioral Sciences and Health Education, Rollins School of Public Health, Emory University, 1518 Clifton Rd.NE, Atlanta, GA 30322, USA; Jingjing.li@emory.edu; 3 School of Public Health and Management, Hubei University of Medicine, 30 South Renmin Road, Shiyan 442000, China; Andyook@21cn.com; 4 School of Nursing, Ningbo College of Health Sciences, 51 Xuefu Road, Ningbo 315100, China; royeelove@163.com; 5 Department of Medical Statistics and Epidemiology, Guangdong Key Laboratory of Health Informatics, Health Information Research Center, School of Public Health, Sun Yat-Sen University, 74 Zhongshan Rd.2, Guangzhou 510080, China; liuxiaohan51@163.com (X.L.); sptmg_d@163.com (Y.Z.); haoyt@mail.sysu.edu.cn (Y.H.); 6 Global Health Institute, Wuhan University, 8 South Donghu Road, Wuhan 430072, China

**Keywords:** Chinese health care reform, satisfaction, medical staff, patient, coping strategy, health management and policy

## Abstract

Satisfaction evaluation is widely used in healthcare systems to improve healthcare service quality to obtain better health outcomes. The aim of this study was to measure employee work satisfaction and patient satisfaction status in Wuhan, China. A cross-sectional study was conducted in 14 medical institutions. The final valid sample comprised a total of 696 medical staff and 668 patients. The overall satisfaction levels of medical staff and patients were 58.28 ± 14.60 (10.47–100.00) and 65.82 ± 14.66 (8.62–100.00), respectively. The factors affecting medical staff satisfaction, ranking in sequence from most to least satisfied, were: the work itself, working environment and atmosphere, hospital management, practicing environment, and job rewards. Patient satisfaction factors, from most to least affecting, were ranked as follows: physician-patient relationship and communication, service organization and facilities, continuity and collaboration of medical care, access to relevant information and support, and healthcare and related services, respectively. The overall satisfaction evaluation of medical staff was average. Healthcare policy makers and medical institution management staff should focus on job rewards and working environment. This would allow them to increase their work happiness and sense of belonging, which in turn would allow them to provide better medical services to patients. The overall patient evaluation was satisfactory, with patients satisfied at all levels of the satisfaction evaluation.

## 1. Introduction

For a long time, work satisfaction has been a core concept of organizational behavior and has been used to measure the attitudes of proactivity or happiness towards work in employees, based on employees’ evaluation of work characteristics (work experience). Furthermore, work satisfaction is an important marker of whether or not a corporation’s reward mechanism is successful [1]. The work satisfaction of medical staff (doctors, nurses, medical technicians, etc.) is related to healthcare service quality and outcomes. Low satisfaction is a major cause of psychological and social stress in medical staff and correspondingly of employee turnover [2,3,4,5]. Many studies have demonstrated that healthcare staff with high satisfaction provide higher quality medical services, resulting in better healthcare outcomes and higher patient satisfaction [6,7,8,9]. On the other hand, patient satisfaction comprises patients’ overall evaluation of the entire healthcare service process and consultation environment, and is a commonly used and key indicator for evaluation of healthcare service quality [10,11]. Analysis of patient satisfaction survey feedback results can provide healthcare workers with sufficient understanding of factors and areas that require improvement and can lead to effective improvements in medical service quality [12,13]. Systematic studies have verified that the medical service quality indicator is the determining factor that has the most influence on patient satisfaction. Another indispensable determining factor is healthcare providers’ interpersonal care quality [14]. At the same time, healthy interactions between medical staff and patients are extremely important and can improve both the physician-patient relationship [15] and nurse-patient relationship [16]. This can encourage medical staff to improve their work efficiency and medical service quality [17] and can also promote patient recovery [18]. In China, tense physician-patient and nurse-patient relationships, insufficient mutual communication, and poor understanding are important factors causing dissatisfaction.

Currently, there are many tools to measure medical staff satisfaction and patient satisfaction worldwide [19,20]. Each tool has its own characteristics, target population, and usage focus, and they are not completely uniform. Tools for measuring work satisfaction in medical staff include universal scales (such as those used for all service staff [21], healthcare service staff [17,22], etc., and specific scales (such as those used for hospital doctors [23,24], hospital nurses [25], dentists [26], nursing home nurses [27], etc.). The measurement populations for these scales may be broad or have a finer professional classification [19]. Tools for measuring patient satisfaction also have universal scales (such as those used to measure patient satisfaction in relation to the healthcare service system [28,29,30], or the satisfaction of hospitalized patients [31]) and specific scales (such as patients’ satisfaction with pharmacy staff [32], nurses [33], etc.). The category of measurement tools for patient satisfaction is not as diverse as that of medical staff work satisfaction. An example is the measurement tool of patients’ satisfaction with pharmacy staff, which was modified from the measurement tool of patients’ satisfaction with healthcare system services [32]. Thus, it can be seen that each satisfaction measurement tool has its design intention for use in accordance with the study objective.

China’s “New Medical Reform”, which began in 2009, is currently ongoing. The satisfaction of all parties regarding healthcare reform can directly reflect the implementation and effect of various measures for medical reform. Investigation tools for medical staff work satisfaction and patient satisfaction have not been combined with China’s new healthcare reforms, as the questionnaire items are not focused on investigating satisfaction with healthcare reform policies and cannot be used to directly investigate satisfaction following the healthcare reform. Research tools will often use foreign measurement scales or scales that were developed before 2009. The five reform priorities of the new healthcare reform are as follows: first, to speed up the construction of the basic medical security system, achieve full coverage and gradually raise the level of funding and security; second, to establish a national basic drug system so that the masses can use drugs safely and effectively; third, it is necessary to improve the basic public health service system, achieve full coverage of the network, change the operational mechanism, and improve the service quality; fourth, to promote the equalization of basic public health services, improve the system and the funding guarantee system, and reduce the basic public health services for urban and rural residents; fifth, to promote a pilot reform of public hospitals, strengthen hospital management, optimize service processes, standardize the diagnosis and treatment activities, improve the level of medical services, and facilitate the public in seeking medical treatment. Therefore, there is a need to develop a scale that is focused on patient satisfaction and medical staff work satisfaction following the healthcare reform. A survey on satisfaction with the new healthcare reform was previously carried out in Guangzhou in December 2014 [34,35] and thereafter promoted in different regions in China. Measurement tools that were verified to be effective through preliminary testing in Guangzhou included, medical staff work satisfaction scales, and patient satisfaction scales comprised the tools used in this study. The survey developed for the present study can closely reflect the current status of patient and medical staff satisfaction after healthcare reform, and can subsequently be used to inspect the implementation status and outcomes of various measures in healthcare reform and propose corresponding policy recommendations.

Previous studies have mostly focused on asking study subjects to describe their level of satisfaction (either medical staff or patients) and thus have segregated medical staff and patients, who should, in fact, be a unified whole and mutually provide feedback and respond to healthcare system operations. This study appropriately expands the research horizons to investigate the status of medical staff work satisfaction and patient satisfaction following China’s healthcare reform in Wuhan (one of the largest cities in China, with abundant healthcare resources) and viewed doctors and patients as a whole in conducting the research.

## 2. Materials and Methods

### 2.1. Study Sample

The subjects of this study comprised medical staff and patients in public general hospitals and community healthcare service centers/stations (medical institutions that are implementing new health reform policies) in Wuhan City, China. The inclusion criteria for medical staff were: 1. specialists, general practitioners, nurses, medical technicians (pharmacists, radiologists, and medical technologists), public health physicians, and hospital management; 2. who have worked at the medical institution for more than half a year; 3. who signed the informed consent form for this study. Exclusion criteria: Medical staff that were in their probation period. Patient inclusion criteria: 1. Patients aged ≥18 years and their relatives; 2. Patients who had attended for diagnosis and treatment in the given medical institution in the past one year (or this round of diagnosis and treatment has already ended); 3. Inpatient and outpatients were both allowed to participate. Exclusion criteria: 1. Non-patient (e.g., scalpers, migrants); 2. Patients who have just arrived at the hospital and have lost touch with the medical staff access to any diagnosis and treatment.

Based on the level of economic development in the administrative region and hospitals that meet a certain scale (integrated public hospitals that can survey at least 65 patients and 65 medical staff and community healthcare service centers/stations that can investigate at least 20 patients and 20 medical personnel), we randomly selected four pilot district-level/municipal-level integrated public hospitals (three tertiary hospitals and one secondary hospital) and 10 community healthcare service centers/stations (10 primary hospitals). At this stage, the data collected were mainly used to evaluate patient satisfaction and medical staff work satisfaction in 2016 in Wuhan City following the implementation of the healthcare reforms. This study adopted stratified sampling to ensure maximal consideration of sampling representation. A total of 720 questionnaires were distributed to medical staff and 680 questionnaires to patients, for a total of 1400 questionnaires. A total of 696 medical staff questionnaires and 668 patient questionnaires were deemed valid for analysis. Twenty-four medical staff questionnaires and 12 patient questionnaires were ultimately deemed invalid. Missing responses were those with blank space or vague to see. Response rate: 96.67% for medical staff and 98.23% for patients. Participants were given a small incentive (a piece of soap or a towel per responder) valued at 5 RMB as compensation for their time. Based on our calculation, the sampling error for medical staff and patient is 3.6% and 3.8%, respectively.

Trained and qualified investigators conducted this study and distributed unified questionnaires. Self-completion methods were mainly used for the collection of questionnaire data in both the medical staff and patient questionnaires. Once informed consent had been obtained from participants, the investigator distributed a questionnaire to them for completion by themselves. Upon completion, the questionnaires were collected and examined for missing items. Throughout the entire study process, the study subjects could ask the investigators if they had any questions or doubts.

To ensure the successful implementation of this study and research quality, rigorous quality control measures were implemented at every stage of the study. A series of measures was used to ensure that differences between the data obtained in this study and the actual situation were kept to a minimum. Quality control was implemented throughout the entire study process: 1. Sampling stage: Comprehensive consideration of sample representativeness and operable design of the sampling protocol, and strict implementation of sampling according to the sampling protocol. 2. Uniform training of investigators and ensuring that the investigators accurately understood the scale dimensions and items. Investigators had to be clear on their roles and responsibilities and have uniform accurate understanding of the questions in the scale and method of answering enquiry questions (non-guided). Medical staff was provided with assistance as needed, including explanation of the meaning of items or skip patterns. 3. Strict implementation of inclusion and exclusion criteria. 4. Establishment of a verification system for survey quality: The investigator must carry out a complete inspection of the content completed by the subject (patient) on site. Investigators would remind the participants that they might forget several items. If the participants suggested they did not wish to answer, then the investigators would respect their decision. 5. To ensure the quality of data entry, a dual entry system was used during the data entry stage, which comprised each questionnaire being independently entered by two investigators. After the data had been verified to ensure no missing items or errors, they were used for statistical analysis. 6. Management of missing values: If missing items were found in some questionnaires during the second review, imputation is necessary. The management method (after discussion with experts) was as follows: If the number of missing values in one questionnaire was >4, the questionnaire was deemed invalid; If the number of missing values in one questionnaire ≤3, such questionnaires were still deemed valid for statistical analysis. Estimates means, standard deviations, covariances, and correlations for different missing value methods: listwise, pairwise, regression, or expectation-maximization (EM). After implementing Little’s MCAR test, it was found that medical staff and patient data belonged to missing at random (MAR) and missing completely at random (MCAR), respectively. Thus, EM and regression method were used for completion of medical staff and patient data, respectively.

### 2.2. Instrument

The study tools comprised two parts:

#### 2.2.1. Medical Staff Work Satisfaction Scale after Healthcare Reform and Patient Satisfaction Scale after Healthcare Reform

The medical staff satisfaction scale following healthcare reform (MSSS-HR, hereinafter referred to as MSSS) and patient satisfaction scale following healthcare reform (PSS-HR, hereinafter referred to as PSS) [34,35] were previously created by the research group based on a literature review of local and overseas measurement tools for medical staff work satisfaction and patient satisfaction and their influencing factors. These were combined with an emphasis on the 2016 national in-depth healthcare system reform to determine the scale dimensions and refine the entry pool. The entry pool was then expanded through two core group interviews. Delphi expert consultation was used to screen the entries in order to generate a preliminary version of the scale. Finally, on-site pre-investigation was used for quantitative evaluation of psychological characteristics. A final improvement was implemented based on the evaluation results to obtain the final scales. MSSS and PSS are progressively used to measure medical staff work satisfaction and patient satisfaction, respectively.

The medical staff work satisfaction scales include five dimensions and 43 items. The five dimensions are: the work itself (first dimension), job reward (second dimension), hospital management (third dimension), work conditions and atmosphere (fourth dimension), and practicing environment (fifth dimension). Satisfaction from the work itself was used to evaluate the satisfaction status of medical staff towards workload, job autonomy, job stability, job importance, ability utilization, job achievement, and job responsibilities. Job rewards were used to evaluate the satisfaction of medical staff towards material rewards, mental rewards, and reward fairness. Hospital management was used to evaluate the satisfaction status of medical staff towards the organization and management of their medical institution, including hospital leadership ability, process management in the hospital, system management, current development status, industry status and developmental outlook, etc. Work conditions and atmosphere were derived from the internal working environment of medical staff and used to evaluate the satisfaction status of medical staff towards the facilities and equipment, informatization level, logistical support, interpersonal relationships and collaborative atmosphere, etc. Practicing environment was derived from the external environment of practice by medical staff and evaluates the satisfaction level of medical staff in relation to physician-patient relationship, communication, societal opinion, health policies, etc. In addition, a special item, with 0 to 100 points description, was included at the end of the medical staff work satisfaction questionnaires to evaluate the macro-level work satisfaction of the study subject in the past year (hereinafter referred to as Special Item-MSSS).

The patient satisfaction scales include five dimensions and 29 items. The five dimensions were: physician-patient relationship and communication (first dimension), access to relevant information and support (second dimension), healthcare and related services (third dimension), continuity and collaboration of medical care (fourth dimension), and service organization and facilities (fifth dimension).The physician-patient relationship and communication dimension mainly refer to the level of satisfaction of the patient towards two-way communication during the consultation process. The physician-patient relationship refers to the social interaction process of mutual communication and interaction between physicians and patients. This is a unique interpersonal relationship, with healthcare at its core and maintenance of patient health as its objective. The access to relevant information and support dimension refers to the process by which patients obtain relevant information in relation to medical services and medical insurance from medical staff. The healthcare and related service dimension refers to the services of examination, diagnosis, treatment, recuperation, and preventive healthcare provided by the medical institution to the patients, as well as the drugs, medical equipment, ambulances, and hospital wards that are related to those services. The continuity and collaboration of the medical care dimension refers to the process, design, and implementation of connecting patients to hospital services in order to provide continuous medical services and coordination between medical staff, in order to ensure that the patient’s medical information is transferred when the patient is transferred, to ensure continuous medical services when patients are transferred to another department or hospital, or following discharge. The service organization and facilities dimension refers to access to medical care and evaluation of perception towards environment and facilities. In addition, a special item, with 0 to 100 points description, was included at the end of the patient satisfaction questionnaire to evaluate the macro-level patient satisfaction towards medical services in the previous year (hereinafter referred to as Special Item-PSS).

The Likert 5-point evaluation method was used for the response scale of each item, with 1 = extremely dissatisfied, 2 = dissatisfied, 3 = generally satisfied or not, 4 = satisfied, and 5 = extremely satisfied, respectively. Another option 6 was also included, which indicated that the respondent “does not understand” the situation; this was converted into an initial value during statistical analysis. The Likert 5-point scale was converted to 0–100 points. Namely, The five-point value range of the standard satisfaction scale was then converted to 0–100 using the following equation [36]:

(1) $$adjSS=\frac{100\left(stdSS-1\right)}{5-1}$$

where, “*adjSS*” and “*stdSS*” represent the “adjusted satisfaction score” and “standard satisfaction score” respectively.

Using a new scoring method, the satisfaction evaluation criteria were developed (adjSS shows the final score of the evaluation system and represents the adjusted satisfaction score): “extremely dissatisfied” (adjSS: 0–), “dissatisfied” (20–), “generally satisfied or not” (40–), “satisfied” (60–), and “extremely satisfied” (80–100).

#### 2.2.2. Self-Designed General Information Questionnaire

The content of the medical staff work satisfaction includes the class of medical institution, gender, age, practice period, educational level, job title, nature of job, employment status, whether at resident/general physician training stage, etc., in the form of multiple choice questions.

The content of the patient questionnaire includes the class of medical institution, the gender, age, household registration status, employment status, educational level, average monthly family income, medical insurance type, whether or not they purchased commercial insurance, consultation type, consultation department, and self-perceived disease severity of the patients or relatives, in the form of multiple-choice questions.

### 2.3. Statistical Analysis

Microsoft Excel (version 2010, Microsoft, Seattle, WA, USA) and EpiData (version 3.1, Lauritsen JM & Bruus M, Odense, Denmark) were used to create a database for data entry. IBM SPSS Statistics (version 18.0, SPSS Inc., Chicago, IL, USA) was used for analysis. Statistical analysis included descriptive analysis, Pearson correlation coefficient, t-test, F-test, multiple linear regression, etc. (the data all conformed to the conditions for statistical testing, such as normality testing). Outcome (dependent) variable is actually only the macro-level work satisfaction for the medical staff (The Special Item-MSSS scoring) and the macro-level patient satisfaction towards medical services for the patient (the Special Item-PSS scoring). Before carrying out multiple linear regression analysis, dummy variable quantitation was carried out on independent variables or predictor variables (medical staff general information, patient general information) to avoid collinearity of independent variables. The tests were all two-tailed tests and *p* < 0.05 or 0.01were deemed statistically significant.

### 2.4. Ethics Statement

Before implementing the study, the ethics committee of Wuhan University School of Medicine (WUSM) reviewed it and found it to comply with the Declaration of Helsinki and its revised versions, as well as the relevant regulations of biomedical journals, and approved the research.At the same time, approval was sought from the relevant management departments of the study units and from medical staff and patients. All study subjects signed an informed consent form. Please see the uploaded annex for the ethics approval form.

## 3. Results

The Cronbach’s alpha of the medical staff work satisfaction scale was 0.973 and the Cronbach’s alpha of each dimension (work itself, job reward, hospital management, work conditions and atmosphere, and practicing environment) were 0.880, 0.860, 0.921, 0.892, and 0.963, respectively. The Cronbach’s alpha of the patient satisfaction scale was 0.949 and the Cronbach’s alpha of each dimension (physician-patient relationship and communication, access to relevant information and support, healthcare and related services, continuity and collaboration of medical care, and service organization and facilities) were 0.871, 0.843, 0.888, 0.641, and 0.878, respectively. The overall reliability of the two scales is thus excellent. Besides continuity and collaboration of medical care (as a dimension, reliability is acceptable), the various dimensions and entire scale all showed ideal and extremely ideal quantitative psychometric characteristics [37,38]. Our study indicates that the correlation coefficients of the dimensions of the medical staff work satisfaction scale after healthcare reform (MSSS) and of the entire scale were 0.775, 0.864, 0.921, 0.884, and 0.888, respectively; the correlation coefficients of the various dimensions and entire scale of patient satisfaction scale after the healthcare reform (PSS) were 0.699, 0.828, 0.917, 0.826, and 0.881, respectively. Research in Guangzhou shows that concerning the MSSS, the Spearman correlation coefficient for each item and its own dimension was between 0.70 and 0.92. Most correlation coefficients between items and their hypothesized subscales were higher than those with other subscales. Five factors emerged from the scale. The cumulative variance contribution rate was 71.04% [34]; as regards the PSS, the scale was composed of 5 dimensions, which could explain 88.223% of overall variance of the scale. The correlation coefficients between the domain scores and the total score of the scale ranged from 0.881 to 0.937 [35]. The validity of the medical staff work satisfaction scale and patient satisfaction scale in Wuhan after healthcare reform were generally consistent with the pre-investigation in Guangzhou. Table 1 shows the general situation. The measurement results of this survey were stable and the measurement tools have good validity and reliability.

### 3.1. Medical Staff and Patient Satisfaction Status

Medical staff satisfaction ranking, in sequence from most to least satisfied was: work itself, work environment and atmosphere, hospital management, practicing environment, and job rewards. Among samples of medical staff, the highest point is 100.00 and the lowest point is 10.47. The correlation coefficient r of overall work satisfaction and the scoring to evaluate the macro-level work satisfaction in the past one year by a special item (the Special Item-MSSS scoring) was 0.605 (*p* < 0.001). The Special Item-MSSS scoring scored 81.45 ± 13.24 and was evaluated as extremely satisfactory.

Patient satisfaction ranking, in sequence from most to least satisfied was: physician-patient relationship and communication, service organization and facilities, continuity and collaboration of medical care, access to relevant information and support, and healthcare and related services. Among samples of patients, the highest point is 100.00 and the lowest point is 8.62. The correlation coefficient r of overall patient satisfaction and the scoring to evaluate the macro-level patient satisfaction towards medical services in the past 1 year by a special item (the Special Item-PSS scoring) was 0.643 (*p* < 0.001). The Special Item-PSS scoring has scored 81.18 ± 13.02 and was evaluated as extremely satisfactory. Medical staff and patient overall satisfaction were evaluated as average and satisfied, respectively. Table 2 and Table 3 show the medical staff and patient satisfaction status.

### 3.2. General Demographic Characteristics and Satisfaction Status of Medical Staff and Patients

Differences were found in the overall satisfaction levels of medical staff in terms of gender, age, educational level, job title, nature of the job, employment status, and whether staff are at the standardized training stage (*p* < 0.05 or 0.01). The class of medical institution approached statistical significance (0.1 > *p* > 0.05). (Table 4). There was no statistically significant difference in various demographic variables in overall patient satisfaction (*p* > 0.05). Household registration status, type of medical insurance, and consultation type approached statistical significance (0.1 > *p* > 0.05). (Table 5).

### 3.3. Multiple Linear Regression Analysis

In Table 6, the macro-level work satisfaction for the medical staff (The scoring Special Item-MSSS) was a dependent variable, while the five dimensions in the medical staff work satisfaction scale and demographic characteristics were independent variables (after the addition of dummy variables). The results of multiple linear regression were that hospital management, practicing environment, work itself, regular training, age, nature of job, and employment status showed statistical significance (*p* < 0.05 or 0.01), can be included in the regression formula, and explain 39.3% of the variance.

In Table 7, the macro-level patient satisfaction towards medical services for the patient (The scoring Special Item-PSS) was a dependent variable and the five dimensions in the patient satisfaction scale and demographic characteristics were independent variables (after the addition of dummy variables). 

The multiple linear regression results found that healthcare and related services, service organization and facilities, access to relevant information and support, educational level, average household monthly income, type of medical insurance, self-perceived disease severity, and hospital class showed statistical significance (*p* < 0.05 or 0.01), can be included in the regression formula, and explain 42.3% of the variance.

## 4. Discussion

### 4.1. Medical Staff Satisfaction Status

The overall work satisfaction for medical staff was average and the scoring to evaluate the macro-level work satisfaction in the past one year by a special item (the Special Item-MSSS scoring) was extremely satisfactory. For work itself and work environment and atmosphere, the satisfaction evaluation results were satisfactory. For job rewards, hospital management, and practicing environment, the satisfaction evaluation results were average. The above conclusion was consistent with survey results from other provinces in China (such as Guangdong, Anhui, Xinjiang, Chongqing, etc.) [36,39,40]. Against the background of “new healthcare reforms”, medical staff have a higher social status, stronger job achievement, and sufficient ability to perform their job. At the same time, the improved hospital work environment has created a better work atmosphere and stimulated job creativity in medical staff, resulting in greater satisfaction in relation to the work itself and hospital environment. However, in terms of job rewards, some hospitals in China continue to adopt the former personnel allocation system, while salaries were allocated according to years of experience, and this does not adequately reflect the job value of medical staff. This resulted in medical staff feeling that job rewards were not in line with their efforts and did not meet their expectations [41]. Optimization of the reward system and improvement of the salary allocation system: An internal, scientific, and suitable method should be used to determine the contributions and value of staff so that staff will feel that the salary system is reasonable. The establishment of a competitive salary system, and ensuring that salary levels are consistent with external markets, will also give medical staff a feeling of fairness. Currently, international medical institutions have begun to explore new salary allocation models, such as the balanced score card theory, which is a healthcare system using a performance-based salary system [42], as well as the use of a resource-based relative value scale (RBRVS) evaluation system to ensure reasonable payment of physician service fees (remuneration payment method) [43], which are useful as a reference for local hospitals. In terms of hospital organization and management, leadership style has a subtle influence on medical staff satisfaction, and management staff should select the optimal leadership style according to the hospital culture and maturity of the staff in respect to the organization [44]. For the practicing environment, medical staff were not very satisfied and there are many reasons for this: tense physician-patient relationships, external public pressure (sometimes the media were misled and did not fairly report medical malpractice, violence towards doctors, rumors, etc.), changes in health policies (resident/general physician training, multiple licenses), etc., particularly violent incidents in the medical institution, and healthcare disturbance [45], which occurs occasionally. These can result psychological problems in medical staff, which can affect their quality of life [46], cause them to be dissatisfied with the practicing environment, and decrease work satisfaction [47]. In addition, in communication, as the party with greater information, when medical staff execute their notification roles, there is a need to be detailed in explaining their job, maintain healthy communications with the patient, and place the patient at the center (through empathy, communication skills, time and information sharing, etc.), in order to alleviate tense physician-patient relationships [48,49]. Macroscopically, medical staff in Wuhan city were satisfied with their work in the last one year and this is largely due to a positive attitude towards work.

In terms of demographic characteristics, this study found that the level of satisfaction was relatively higher in females and this was consistent with a number of previous studies in China [36]. However, other studies have also found that male gender is a positive influencing factor for work satisfaction in medical staff [50], while still others found no differences between male and female medical staff in terms of satisfaction [51,52]. Therefore, further study on the effects of gender on work satisfaction is required. For age, there was a “U-shape distribution” of satisfaction with ages 40–49 years showing the lowest level of satisfaction. This may be because medical staff in this age group may be at their peak of their career and have “professional plateau” reactions, which manifest as significant burnout [53]. Medical staff with educational levels of graduate student and above had the lowest level of satisfaction. This is because highly educated medical staff have higher qualifications and technical expertise, and as a result have more job responsibilities and stress in their departments. Medical staff with no job title or junior medical staff have a higher level of satisfaction, while intermediate and senior medical staff have a lower level of satisfaction. This result is identical to the results of studies in Anhui and Xinjiang provinces [36]. Staff with no job title and junior staff have only recently entered the workforce or have been working for a short period of time. In addition, as they have little work experience, factors such as promotion, treatment, training, and learning will create negative effects on work satisfaction. However, intermediate and senior medical staff are the backbone of medical institutions and have greater work intensity and occupational risks, bear more responsibilities, and face greater burnout and psychological stress. This results in low work satisfaction in this group. In terms of job nature, medical technicians and management staff have higher satisfaction levels compared with physicians and nurses. This is because they have regular rest periods (generally, they start and finish work on time, with little overtime) and have no direct contact with patients, leading to a higher level of satisfaction. Nurses have a higher level of satisfaction than physicians as, under the Chinese healthcare system, physicians are the leaders in diagnosis and treatment, while nurses play a subordinate role in these tasks (physicians have the authority to prescribe drugs but not nurses [54]). Thus, nurses face fewer risks and less stress than physicians. Contract medical staff and medical staff that are undergoing standardized training have a higher level of work satisfaction. This is because they have lower educational levels, experience, or technical expertise, and value their current positions. They also have lower expectations and these results in greater work satisfaction. In addition, the difference in the level of satisfaction between medical staff in different types of medical institution approached statistical significance. Medical staff in secondary and tertiary hospitals (district/municipal public hospitals) have higher levels of satisfaction compared with primary hospitals (community healthcare service center/stations) as higher-class hospitals have a better work environment and conditions.

### 4.2. Patient Satisfaction Status

This study showed that overall patient satisfaction evaluation under the “new healthcare reform” was satisfactory, and that the 5-dimension evaluation was satisfactory. Of the five dimensions, although patients were satisfied with healthcare and related services (barely satisfied), this factor ranked the lowest. The scoring to evaluate the macro-level patient satisfaction towards medical services in the past 1 year by a special item (the Special Item-PSS scoring) was also very satisfactory. This reflects the effectiveness of reform initiatives in the “new healthcare reform” policy towards shortcomings that have been present for a long time in the healthcare domain and has reversed the damage in market orientation towards the healthcare domain. The reforms have resulted in a return to fairness and welfare in basic healthcare services [55], and has secured gratifying progress and breakthroughs and fostered widespread approval and recognition from patients.

In terms of demographic information, the levels of medical insurance of inpatients and outpatients in Wuhan City was 94.9%, and this has achieved the goal of the basic medical insurance system, covering 90% of urban and rural residents. This demonstrates that the national public health insurance scheme has gradually taken shape. In recent years, with the continuous expansion of medical insurance coverage, medical insurance payment levels have increased, and the rapid growth of per capita healthcare costs have been controlled to some extent. Government, social, and individual healthcare expenditure are basically balanced and can reasonably share healthcare costs [56]. In addition, it has been found that the differences in overall satisfaction between patients with different types of medical insurance approached statistical significance: Patients with no medical insurance (self-paid) have the highest level of satisfaction. This may be because the majority of these patients have better financial capabilities or are in a better state of health, have stronger immunity, or less severe symptoms. This was not in line with conventional thinking that patients with medical insurance have a higher level of satisfaction [57]. Some studies found that patients with urban occupational health insurance have a higher level of satisfaction [58]. The greatest challenge faced by China’s new healthcare reform is in relation to how medical insurance institutions can implement effective constraints and controls on medical institutions so that the latter can provide cost-effective medical services to insured patients and ultimately ensure universal public health insurance. Differences in household approached statistical significance and foreign populations have a higher level of satisfaction than local populations. This is because the majority of foreign populations were service workers from areas around Wuhan, and healthcare in their hometown may not be as good as the healthcare conditions and environment in Wuhan. In terms of patient type, their differences approached statistical significance as inpatients have a higher level of satisfaction than outpatients. Generally, inpatients undergo outpatient consultation and have a longer contact time with the medical institution and medical staff (outpatients have shorter consultations times and their conditions are often less serious) and can better experience medical services during the treatment process, resulting in increased satisfaction.

## 5. Limitations and Suggestions for Future Research

Although our investigators have carried out in-depth exploration of satisfaction in medical staff and patients and analyzed several factors, we believe that the study has some shortcomings (e.g., Because the research objects are too busy, only fewer emergency medical staff and patients included in this study), with factors that were difficult to estimate excluded from the study (such as living state, marital status, the patient’s objective condition, etc.). We hope that in the future, we can include more variables (factors) that affect medical staff work satisfaction and patient satisfaction for a further in-depth study:

1 The study sample only comprised 14 medical institutions in one major city (Wuhan) in China. We did not include other cities in China to obtain a larger sample because different regions have different economic progress and cultural differences, which may affect satisfaction. We can focus on certain sections of the responder satisfaction in future research, like pre-hospital emergency [59].
2 The cross-sectional design of this study limited the inference of causal relationships and was time based. The study tools were only tested in Guangzhou and Wuhan. Although the effectiveness of the tools was initially verified, more samples from different regions and medical institutions are required for continuous measurement and verification in order to optimize and improve the measurement tool.
3 While the data presented in this study were quantitative, at the same time, qualitative descriptive data were also collected from medical staff and patients (through panel discussions, etc.). Due to the limitations on manuscript length, we will carry out more in-depth verification and comparison of qualitative data in the further study.

## 6. Conclusions

This study has shown that the overall satisfaction of medical staff is affected by many variables, including those found to be statistically significant in the previous sections. However, satisfaction is not only limited to these factors. Although patient satisfaction did not show any statistically significant differences in the demographic data of enrolled patients, some variables still approached statistical significance and deserve further study for verification.

On the whole, the overall satisfaction evaluation of medical staff was average. Healthcare policy makers and the management of medical institutions should focus on the aspects of job rewards and practicing environment in order to decrease the risk of dissatisfaction in medical staff and increase their work happiness and sense of belonging so that they can provide better medical services to patients. The overall patient evaluation was satisfactory and patients were satisfied in all levels of the satisfaction evaluation. However, further improvements and enhancements should be made in healthcare and related services.

## Figures and Tables

**Table 1:** Comparison of Cronbach’s alpha of the MSSS and PSS between Wuhan and Guangzhou.

	MSSS		PSS	
	Wuhan	Guangzhou	Wuhan	Guangzhou
Scale	0.973	0.98	0.949	0.98
Dimension1	0.880	0.90	0.871	0.93
Dimension2	0.860	0.89	0.843	0.88
Dimension3	0.921	0.92	0.888	0.86
Dimension4	0.892	0.89	0.641	0.86
Dimension5	0.963	0.95	0.878	0.83

**Table 2:** Medical staff satisfaction and evaluation.

Dimension	No. of Items in Dimension	Adjusted Satisfaction Score	Satisfaction Evaluation	Ranking
Overall work satisfaction	43	58.28 ± 14.60	Average	-
Work itself	8	63.03 ± 13.96	Satisfactory	1
Job rewards	7	54.06 ± 16.35	Average	5
Hospital management	8	58.53 ± 17.38	Average	3
Work environment and atmosphere	8	62.97 ± 15.35	Satisfactory	2
Practicing environment	12	54.28 ± 19.31	Average	4

Note: Ranked from highest to lowest.

**Table 3:** Patient satisfaction and evaluation.

Dimension	No. of Items in Dimension	Adjusted Satisfaction Score	Satisfaction Evaluation	Ranking
Overall patient satisfaction	29	65.82 ± 14.66	Satisfactory	-
Physician-patient relationship and communication	3	77.07 ± 17.44	Satisfactory	1
Access to relevant information and support	5	64.66 ± 18.46	Satisfactory	4
Healthcare and related services	9	60.88 ± 17.29	Satisfactory	5
Continuity and collaboration of medical care	3	66.40 ± 17.21	Satisfactory	3
Service organization and facilities	9	67.47 ± 16.00	Satisfactory	2

Note: Ranked from highest to lowest.

**Table 4:** Demographic characteristics associated with the medical staff’s job satisfaction (*N* = 696).

		*n* (%)	adjSS	*p*
Type of medical institution (Class)	District/municipal public hospital (tertiary and secondary)	453 (65.1)	58.96 ± 14.94	0.092
	Community healthcare service center/stations (Primary hospitals)	243 (34.9)	57.00 ± 13.87	
Gender	Male	181 (26.0)	55.76 ± 15.87	0.007 **
	Female	515 (74.0)	59.16 ± 14.03	
Age	18–29	290 (41.7)	59.96 ± 14.59	0.049 *
	30–39	245 (35.2)	57.26 ± 15.08	
	40–49	101 (14.5)	55.62 ± 14.32	
	50–59	52 (7.5)	58.04 ± 12.46	
	>60 years	8 (1.1)	63.74 ± 9.28	
Practicing period	<5 years	271 (38.9)	59.12 ± 14.37	0.590
	6–10 years	166 (23.9)	58.21 ± 15.66	
	11–15 years	97 (13.9)	58.67 ± 15.27	
	16–20 years	42 (6.0)	56.60 ± 14.06	
	Over 20 years	120 (17.2)	56.76 ± 13.23	
Educational level	High school, vocational school, polytechnic and below	28 (4.0)	59.69 ± 12.89	0.015 *
	College or senior technical school	140 (20.1)	58.94 ± 13.08	
	Undergraduate	372 (53.4)	59.32±14.85	
	Graduate student and above	156 (22.4)	54.96±15.19	
Job title	None	63 (9.1)	61.19±16.67	0.003 **
	Junior	367 (52.7)	59.75 ± 13.84	
	Intermediate	202 (29.0)	55.20 ± 14.52	
	Vice-senior	55 (7.9)	56.93 ± 14.96	
	Senior	9 (1.3)	55.06 ± 18.76	
Nature of job	Specialist	177 (25.4)	53.67 ± 14.85	<0.001 **
	General practitioner	41 (5.9)	54.18 ± 15.00	
	Medical technical staff (pharmacist, medical technologist, radiologist, etc.)	42 (6.0)	63.06 ± 13.65	
	Nurse	342 (49.1)	60.86 ± 14.33	
	Public health physician	56 (8.0)	54.04 ± 12.64	
	Management staff	38 (5.5)	61.94 ± 11.23	
Employment status	In-house staff	210 (30.2)	56.26 ± 15.15	0.003 **
	Contract staff	324 (46.6)	60.28 ± 13.76	
	Others (labor dispatch, personnel agency, secondment, retiree, etc.)	162 (23.3)	56.90 ± 15.06	
Whether at resident/general physician training stage	Yes	114 (16.4)	62.28 ± 15.33	0.001 **
	No	582 (83.6)	57.49 ± 14.33	

Note: * *p* < 0.05, ** *p* < 0.01.

**Table 5:** Demographic characteristics associated with the patients’ satisfaction (*N* = 668).

		*n* (%)	adjSS	*p*
Type of medical institution (Class)	District/municipal public hospital (secondary and tertiary)	401 (60.0)	65.74 ± 15.03	0.863
	Community healthcare service center/stations (Class 1)	267 (40.0)	65.94 ± 14.12	
Gender	Male	289 (43.3)	66.69 ± 14.74	0.180
	Female	379 (56.7)	65.16 ± 14.59	
Age	18–29 years	191 (28.6)	66.06 ± 14.79	0.493
	30–39 years	111 (16.6)	63.95 ± 12.19	
	40–49 years	88 (13.2)	65.89 ± 14.82	
	50–59 years	116 (17.4)	67.50 ± 15.84	
	>60 years	162 (24.3)	65.59 ± 15.12	
Household registration status	Local urban resident	346 (51.8)	65.28 ± 14.48	0.074
	Local agricultural worker	65 (9.7)	62.52 ± 15.10	
	Foreign permanent resident	117 (17.5)	67.88 ± 15.01	
	Foreign non-permanent resident	140 (21.0)	66.98 ± 14.39	
Employment status	Government agencies	28 (4.2)	66.44 ± 17.00	0.181
	Enterprises and institutions	155 (23.2)	65.19 ± 15.69	
	Freelancer	131 (19.6)	66.05 ± 14.06	
	Foreign laborer	41 (6.1)	66.57 ± 13.01	
	Unemployed	35 (5.2)	64.54 ± 15.65	
	Student	49 (7.3)	71.78 ± 13.28	
	Retiree	177 (26.5)	64.58 ± 14.41	
	Others	52 (7.8)	65.68 ± 13.78	
Educational level	Primary school and below	40 (6.0)	68.83 ± 12.72	0.209
	Junior high school	133 (19.9)	67.14 ± 12.25	
	Vocational school	71 (10.6)	65.63 ± 14.72	
	High school, polytechnic	115 (17.2)	66.84 ± 13.56	
	College	128 (19.2)	63.34 ± 17.23	
	Undergraduate and above	181 (27.1)	65.37 ± 15.26	
Average household monthly income (RMB)	0~	22 (3.3)	59.46 ± 16.60	0.225
	500~	121 (18.1)	66.32 ± 12.38	
	2000~	271 (40.6)	65.59 ±14.29	
	4000~	184 (27.5)	65.82 ±16.54	
	8000~	70 (10.5)	67.84 ±13.63	
Average household monthly income (RMB)	UEBMI	283 (42.4)	64.81 ± 14.15	0.064
	URBMI	145 (21.7)	67.27 ± 15.18	
	NCMS	158 (23.7)	64.63 ± 13.49	
	Others	48 (7.2)	67.61 ± 16.51	
	None (self-paid)	34 (5.1)	71.08 ± 17.82	
Purchased commercial insurance	Yes	145 (21.7)	65.35 ± 15.26	0.664
	No	523 (78.3)	65.95 ± 14.51	
Consultation type	Outpatient	362 (54.2)	64.89 ± 14.36	0.074
	Inpatient	306 (45.8)	66.92 ± 14.96	
Consultation department	Internal medicine	301 (45.1)	66.78 ± 14.56	0.204
	Surgery	135 (20.2)	66.72 ± 14.99	
	Gynecology	61 (9.1)	65.42 ± 17.23	
	Pediatrics	17 (2.5)	63.44 ± 9.43	
	Others	154 (23.1)	63.57 ± 13.82	
Self-perceived disease severity	Slight	169 (25.3)	65.93 ± 14.04	0.461
	Moderate	317 (47.5)	65.17 ± 14.41	
	Serious	182 (27.2)	66.86 ± 15.66	

Note: UEBMI: Urban Employee Basic Medical Insurance; URBMI: Urban Resident Basic Medical Insurance; NCMS: New Cooperative Medical System.

**Table 6:** Results of multi-factor linear regression analysis for the medical staff’s job satisfaction.

Relative Factors	Partial Regression Coefficients	Standard Error	Standardized Partial Regression Coefficients	T	*p*
Constant	62.072				
Hospital management	0.196	0.039	0.257	4.962	<0.001
Practicing Environment	0.176	0.032	0.256	5.443	<0.001
Work itself	0.154	0.040	0.162	3.844	<0.001
Regular training (Yes, Reference: None)	−2.688	1.191	−0.075	−2.256	0.024
Age (18–29, reference > 60)	−10.276	4.977	−0.383	−2.065	0.039
Age (30–39, reference > 60)	−10.210	4.787	−0.368	−2.133	0.033
Nature of job (medical technician, reference: management)	−4.832	2.372	−0.087	−2.037	0.042
Employment status (in-house, reference: others)	−3.181	1.433	−0.110	−2.220	0.027

Note: *R*^2^ = 0.415, adjR^2^ = 0.393 (F = 18.287, *p* < 0.001).

**Table 7:** Results of multi-factor linear regression analysis for the patients’ satisfaction.

Relative Factors	Partial Regression Coefficients	Standard Error	Standardized Partial Regression Coefficients	T	*p*
Constant	45.834				
Healthcare and related services	0.248	0.041	0.329	6.067	<0.001
Service organization and facilities	0.222	0.038	0.273	5.817	<0.001
Access to relevant information and support	0.073	0.030	0.104	2.405	0.016
Educational level (primary school, reference: undergraduate and above)	4.549	2.155	0.083	2.111	0.035
Average household monthly income (500–2000 RMB, reference: 8000–)	3.830	1.655	0.113	2.314	0.021
Medical insurance type (NCMS, reference: None)	−4.145	2.022	−0.135	−2.050	0.041
Self-perceived disease severity (moderate, reference: serious)	2.330	1.007	0.089	2.315	0.021
Hospital class (secondary and tertiary, reference: primary hospital)	2.254	0.966	0.085	2.332	0.020

Note: *R*^2^ = 0.455, adjR^2^ = 0.423 (F = 14.197, *p* < 0.001).
